# Supplementary material for: MiR-205-driven downregulation of cholesterol biosynthesis through SQLE-inhibition identifies therapeutic vulnerability in aggressive prostate cancer
Source: Nat Commun. 2021 Aug 20;12:5066. doi: 10.1038/s41467-021-25325-9 (PMC8379214; doi:10.1038/s41467-021-25325-9)
Supplement: Supplementary file 1 — Supplementary Information [file 41467_2021_25325_MOESM1_ESM.pdf]

Supplementary Information - Kalogirou et al.

MiR-205-driven downregulation of cholesterol biosynthesis through SQLE-inhibition identifies therapeutic vulnerability in aggressive prostate cancer

Supplementary Note 1: Detailed case descriptions of patients

Patient #1: This patient was diagnosed with metastatic PCa (osseous, Gleason score 8, iPSA 724 ng/ml), but retained a good overall performance status (ECOG 0) (Supplementary Table 2 and Supplementary Figure 8a and b). After a long treatment history, including ADT, radiation, enzalutamide, abiraterone, docetaxel (6x) and an experimental treatment with one cycle of 6.5 GBq Lu-177-PSMA, which lead to a nadir PSA value of 1.2 ng/ml, PSA levels rapidly increased at the beginning of 2019 and exaggerated after cancellation of a second-line cabazitaxel (4x) chemotherapy in May 2019 to a value of 1110 ng/ml (Fig. 2c). During this time, biopsies of liver metastases were taken to determine BRCA mutation status with the aim to include the patient in a PARP-inhibitor (PARPi) trial. We also confirmed efficient testosterone suppression and excluded a larger neuroendocrine tumour component by measurement of serum neurone-specific enolase (NSE) and serum Chromogranin A. After providing informed consent, the patient was treated with oral terbinafine 250 mg twice daily during a window of opportunity before start of the PARPi trial. After two weeks of treatment, PSA had dropped from over 1100 ng/ml to 864 ng/ml (Fig. 8b) without the patient experiencing greater adverse side-effects (> grade III according to CTCAE criteria). Treatment had to be cancelled due to start of the PARPi study. Unexpectedly, the patient died two weeks after terbinafine cessation due to an unrelated cause (pulmonary embolism).

Patient #2: This patient was diagnosed with metastatic PCa (osseous and lymph node, Gleason Score 9, iPSA 251 ng/ml) with poor performance status (ECOG 3) (Supplementary Table 2 and Supplementary Figure 8c and d). The patient was treated with ADT, abiraterone, enzalutamide and palliative docetaxel (6x), after which he progressed and did not qualify for any further treatment in 2019. Nadir PSA values during these treatments were between 1 and 2.1 ng/ml. Laboratory analysis suggested a neuroendocrine component of the disease with a chromogranine A of 1900 ng/ml. The disease accelerated to a PSA value of 894 ng/ml (Fig. S8c). Notably, the patient had received cholesterol-lowering treatment with atorvastatin 40 mg daily for several years. After providing informed consent, the patient received oral terbinafine 250 mg once daily (the dose was reduced due to mild liver impairment) for 2 weeks with no severe side effects. After two weeks of treatment, PSA had dropped from 894 ng/ml to 793 ng/ml (Fig. 8c), after which the treatment was discontinued upon the patient's request. The patient agreed to ongoing PSA measurements and an increase to 903 ng/ml was observed

four weeks after treatment cessation. This patient died of accelerating disease two months after terbinafine treatment termination.

Patient #3: This patient was diagnosed with metastatic PCa (synchronous osseous and adrenal gland, Gleason-Score 10, iPSA 480 ng/ml, Supplementary Table 2). The patient was initially treated with ADT and radiotherapy as first line therapy, which resulted in nadir PSA values of 8 ng/ml. After progression to castration-resistant disease, the patient received docetaxel and cabazitaxel and, thereafter, was treated with abiraterone followed by enzalutamide. After PSA-progression to 200 ng/ml, the patient received 6 cycles of 6.2 GBq Lu-177-PSMA each, after which disease accelerated quickly with a PSA of 1251 ng/ml at four months after LU-177-PSMA treatment. During this time, core biopsies of bone metastases were taken to determine *BRCA* mutation status for inclusion in a PARPi trial. After providing informed consent, the patient was treated with oral terbinafine at a dose of 250 mg twice daily during a window of opportunity before potential inclusion into the PARPi trial. After two weeks of treatment, PSA had risen from over 1251 ng/ml to 1440 ng/ml without the patient experiencing greater adverse side-effects (> grade III according to CTCAE criteria). However, due to the experienced clinical benefit and after joint discussion with the patient, terbinafine treatment was continued for another four weeks. The PSA level after 6 weeks of treatment was determined with 1410 ng/ml. Treatment was then stopped at the patient's request, since cancerous bone marrow infiltration necessitated repeated blood transfusions. Consequently, the therapy was switched to best supportive care and the patient died four months after cessation of terbinafine treatment due to progressive bone marrow infiltration of the tumour.

Patient #4: This patient was diagnosed with metastatic PCa (synchronous lymph node metastases, Gleason-Score 8, iPSA 26.7 ng/ml, Supplementary Table 2). The patient was initially treated with ADT and, after progressing to castration-resistant disease, received 5 cycles of docetaxel. Symptomatic pelvic lymph node metastasis progression with bladder infiltration and macrohematuria was treated with radiation therapy at a PSA level of 75.6 ng/ml. Sequential treatment with enzalutamide and abiraterone resulted in a PSA nadir of 55 ng/ml. The patient was hospitalised two months later with progressive lymph node metastasis and new-onset macrohematuria and a PSA of 321 ng/ml. After providing informed consent, the patient received oral terbinafine at a dose of 250 mg twice daily for two weeks without experiencing greater adverse side-effects (> grade III according to CTCAE criteria). PSA dropped to 281 ng/ml after two weeks and terbinafine treatment was stopped upon the patient's request. The patient died two months after cessation of terbinafine treatment.

Supplementary Tables:

Supplementary Table 1: Cox analysis of SQLE protein expression and clinicopathological and outcome data for two PCa patient cohorts**.** Significant p-values are in bold.

TMA1 recurrence-free survival (RFS)	univariate analysis				multivariate analysis			
variable	HR	low95	high95	p-value	HR	low95	high95	p-value
age (continuous)	1.004	0.9515	1.043	0.874				
preOP PSA (continuous)	1.007	0.9808	1.034	0.6				
gleason score (continuous)	1.559	1.266	1.92	**<0,0001**	1.309	0.9552	1.793	0.0941
tumour stage (pT, continuous)	1.782	1.196	2.655	**0.004**	1.055	0.633	1.759	0.8369
nodal status (pN, categorial)	3.422	1.531	7.647	**0.001**	2.054	0.8716	4.838	0.0998
resection status (R, categorial)	1.12	0.57	2.201	0.7				
SQLE expression (continuous)	1.303	1.173	1.449	**<0,0001**	1.18	1.0214	1.364	**0.0247**
Median SQLE expression (dichotomized)	2.893	1.462	5.726	**0.0014**				

TMA2 recurrence-free survival (RFS)	univariate				multivariate analysis			
variable	HR	low95	high95	p-value	HR	low95	high95	p-value
age (continuous)	1.036	0.9134	1.021	0.2				
preOP PSA (continuous)	1.001	0.9944	1.007	0.8				
gleason score (continuous)	1.122	0.918	1.371	0.3	1.075	0.9069	1.274	0.4043
tumour stage (pT, continuous)	1.126	0.547	2.317	0.7				
nodal status (pN, categorial)	1.115	0.4991	2.49	0.8				
resection status (R, categorial)	1.232	0.4549	3.337	0.7				
SQLE expression (continuous)	1.235	1.046	1.459	**0.01**	1.249	0.9069	1.274	**0.00383**
Median SQLE expression (dichotomized)	2.431	1.09	5.419	**0.03**				

Supplementary Table 2: Clinical characteristics of the PCa patients

**Patient**	**#1**	**#2**	**#3**	**#4**
ECOG at terbinafine start	0	3	3	2
Karnovsky at terbinafine start	80	60	50	70
slope log (PSA)	0.37	0.32	0.43	0.26
PSA velocity pre terbinafinetherapy (ng/ml/month)	129.31	98.44	202.85	31.58
PSA doubling time pre terbinafine therapy (months)	1.86	2.18	1.6	2.7
previous therapies (excluding radiation and ADT)	5	3	5	3
terbinafine dosage (per 24h)	500 mg	250mg	500 mg	500 mg
terbinafine treatment (weeks)	2	2	6	2
PSA pre terbinafine	1100	904	1251	321
PSA 2 weeks post terbinafine start	864	790	1410	288
best change in PSA (% to baseline)	-28.4	-14.4	12.2	-11.4
death after terbinafine treatment (months)	0.5	2	4	2

Supplementary Table 3: Primers used in this study

Gene Name (Symbol)	fw sequence	rev sequence	Procuct name / Supplier	GeneGlobe Id	cat. #
β-Actin (ACTB)	5'- cct ggc acc cag cac aat -3'	5'- ggc gat cca cac gga gta ct -3'	n/a	n/a	
PSA (KLK3)	5'- gtg acg tgg att ggt gct gca -3'	5'- ctg ggg gtg cac cag aac a -3'	n/a	n/a	
TMPRSS2	5'- att ctt gcc agg gtg aca g -3'	5' - cca tca cat tcc cgt aca c -3'	n/a	n/a	
SQLE	n/a	n/a	RT² qPCR Primer Assay for Human SQLE (Qiagen)	PPH06371A-200	330001
Androgen Receptor (AR)	n/a	n/a	Hs_AR_1_SG QuantiTect Primer Assay (Qiagen)	QT00073451	249900
E-Cadherin (CDH1)	n/a	n/a	Hs_CDH_1_SG QuantiTect Primer Assay (Qiagen)	QT00080143	249900
Vimentin (VIM)	n/a	n/a	Hs_VIM_1_SG QuantiTect Primer Assay (Qiagen)	QT00095795	249900
ZEB1	n/a	n/a	Hs_ZEB1_1_SG QuantiTect Primer Assay (Qiagen)	QT00597954	249900
RNU6b	n/a	n/a	TAQMan probe 001093 (Thermofisher)	n/a	4427975
miR-205	n/a	n/a	Hs03302942_pri (ThermoFisher)	n/a	4427012

Supplementary Figures

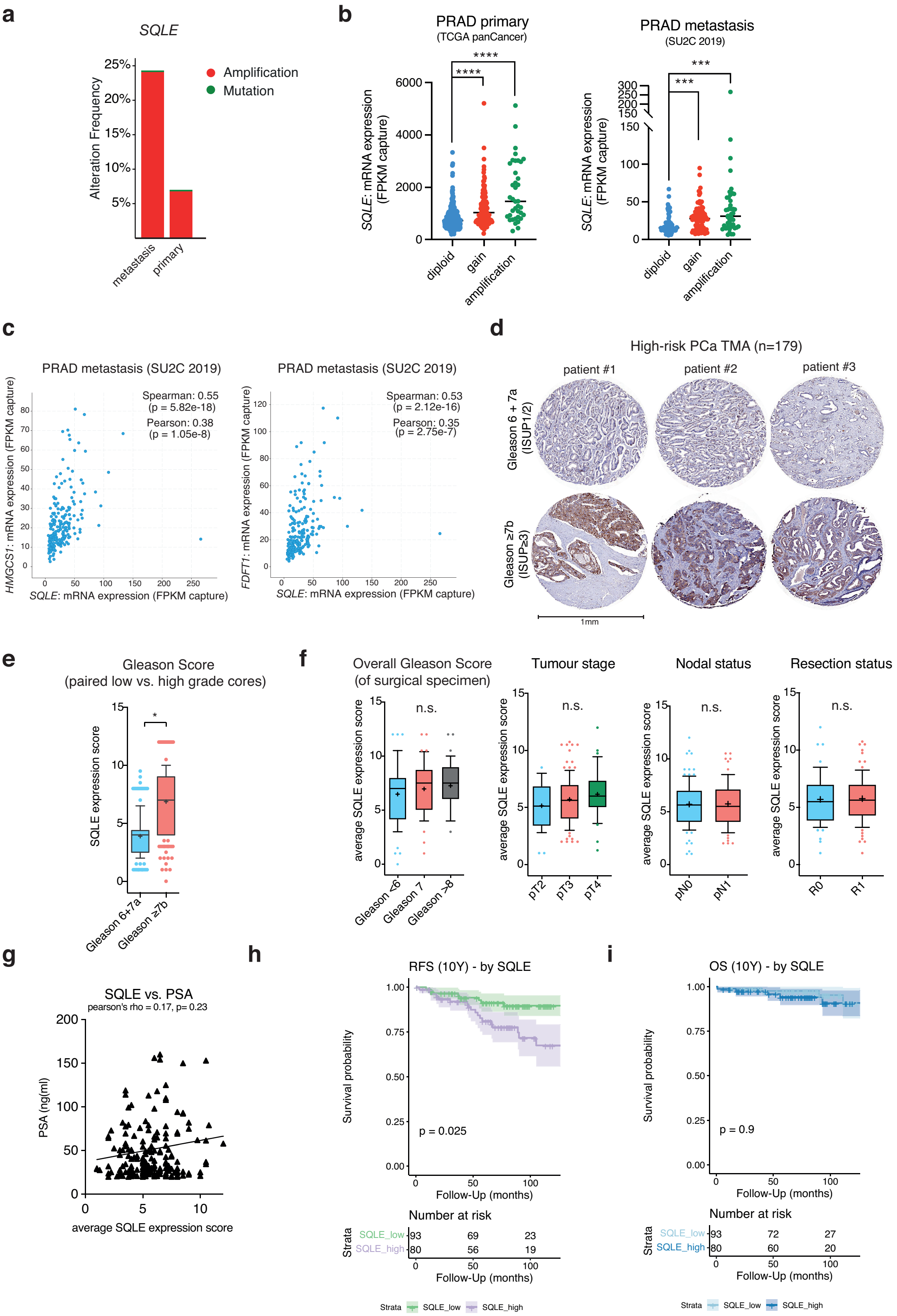

Extended Data Figure 1

Supplementary Figure 1:

a) Percentage of *SQLE* gene alterations (amplification/mutation) in metastatic prostate cancer (SU2C/PCF Dream Team, PNAS 2019, *N* = 444) and primary prostate adenocarcinoma (PRAD) (TCGA, PanCancer Atlas, *N* = 494) determined using the “cBioportal” tool (http://www.cbioportal.org).

b) Expression of *SQLE* in primary and metastatic PCa according to diploid, gain and amplification status. FPKM = fragments per kilobase per million mapped reads. Data are presented as scatter dot plot with line at median (***=p<0.001, ****=p<0.0001, unpaired two-tailed Student’s t-test without multiple testing correction).

c) Pearson correlation (two-sided) of *SQLE* with *HMGCS1* and *FDFT1* expression as assessed by log2 FPKM in a metastatic prostate cancer dataset (SU2C/PCF Dream Team, PNAS 2019, n=444).

d) SQLE expression in representative tissue cores of three patients from a second PCa patient cohort represented on tissue microarray 2 (TMA2) (*N* =179). For each patient, tumour tissue samples displaying medium Gleason score (6+7a, ISUP1-2, top) were compared to tissue samples displaying high Gleason score (≥7b, ISUP≥3, bottom).

e) Box and whiskers plots (box 25-75th percentile, whiskers 10-90th percentile, line at median, + at mean) of paired SQLE expression scores in a grouped analysis of tumour tissue samples with low and high Gleason scores from the same patient of the second PCa cohort (TMA2, *N* =179). (*p<0.05, paired two-sided Wilcoxon signed rank test).

f) Box and whiskers plots (box 25-75th percentile, whiskers 10-90th percentile, line at median, + at mean) of average SQLE expression scores in tumour tissue samples compared by overall Gleason score, tumour stage, nodal status and resection status from the same cohort as in (e). (n.s.= not significant, paired two-sided Wilcoxon signed rank test).

g) Pearson correlation (one-sided) of average SQLE expression scores with pre-operative prostate specific antigen (PSA) levels in patients from the same cohort.

h-i) 10-year Kaplan-Meier survival estimates for recurrence-free survival (RFS) (h) and overall survival (OS) (i) survival in the PCa cohort represented on TMA2 (*N* = 179), dichotomized into high (“SQLE_high”) and low (“SQLE_low”) SQLE expression. Median *SQLE* expression score was used for dichotomization. Survival differences were calculated with the log-rank test.

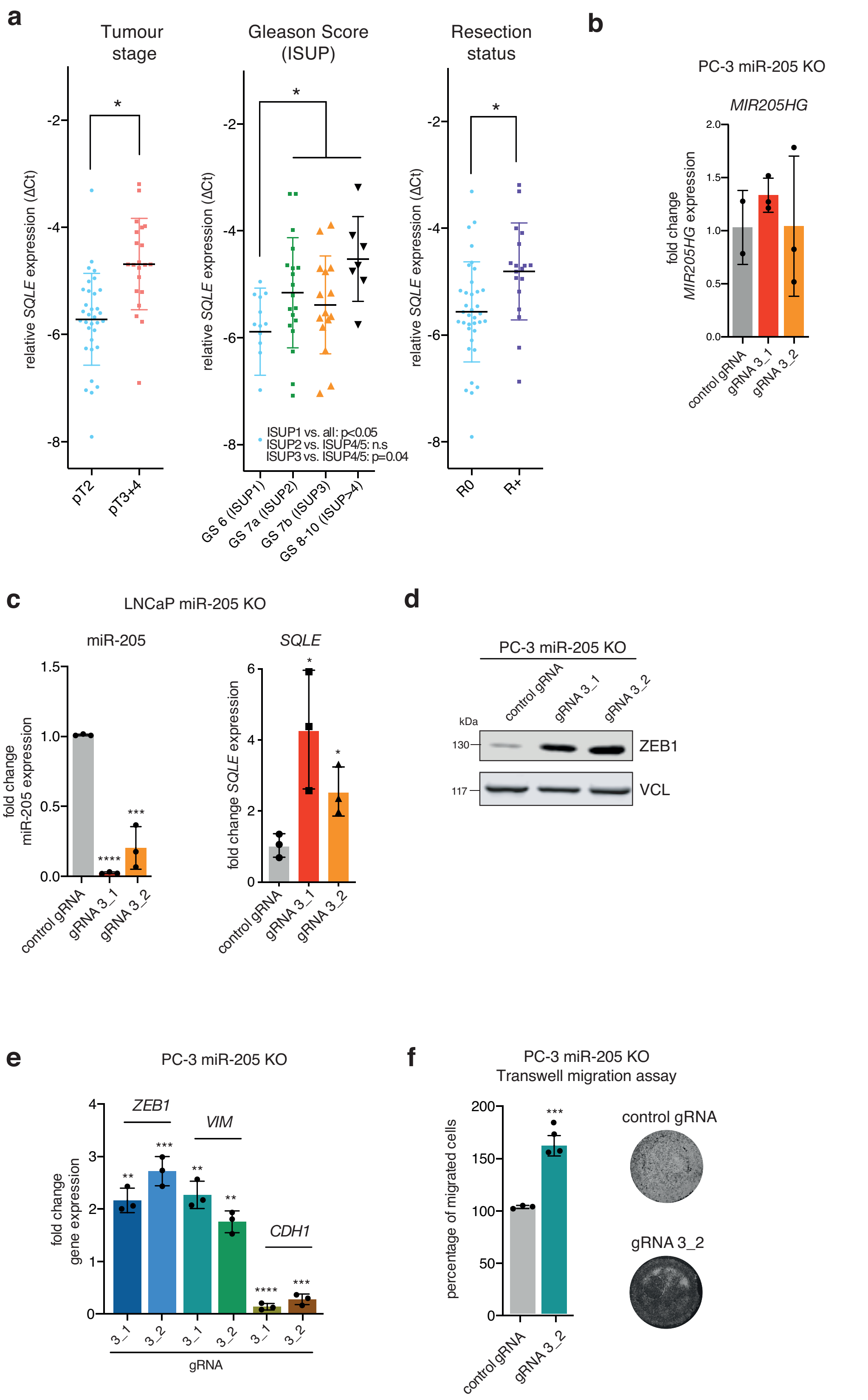

Extended Data Figure 2

Supplementary Figure 2:

a) Relative expression of *SQLE* in an in-house cohort of prostate cancer patients (*N* =59) determined by qRT-PCR and analysed according to tumour stage, Gleason score (ISUP stage) and resection status (R0/R+). Data presented as mean +/- SD (*=p<0.05, unpaired two-tailed Student's t-test).

b) Expression of *MIR205HG* mRNA in pools of PC-3 cells after deletion of miR-205 using two combinations of gRNAs. Cells expressing Cas9 and a non-targeting guide-RNA were used as controls. Data presented as mean +/- SD of three independent samples.

c) Expression of miR-205 and *SQLE* in pools of LNCaP cells containing Cas9 and the indicated combination of two guide-RNAs. Cells expressing Cas9 and a non-targeting guide-RNA were used as controls. Data presented as mean +/- SD of three independent samples (*p<0.05, unpaired two-tailed Student's t-test).

d) WB showing expression of ZEB1 in PC-3 cells either wild type or knock-out for miR-205. Vinculin (VCL) is shown as loading control. A representative image of two independent experiments is shown.

e) Expression of genes associated with epithelial to mesenchymal transition (EMT) by qRT-PCR (*ZEB1*, *VIM* and *ECAD*) in pools of miR-205 knock-out PC-3 cells expressing Cas9 and the indicated combination of two guide-RNAs. Cells expressing Cas9 and a non-targeting guide-RNA were used as controls. Data presented as mean +/- SD of three independent experiments and are normalised to non-targeting controls (*p<0.05, unpaired two-tailed Student's t-test).

f) Transwell migration assay of PC-3 cells either wild type or knock-out for miR-205. Data are presented as mean +/- SD of three independent experiments (*p<0.05, unpaired two-tailed Student's t-test).

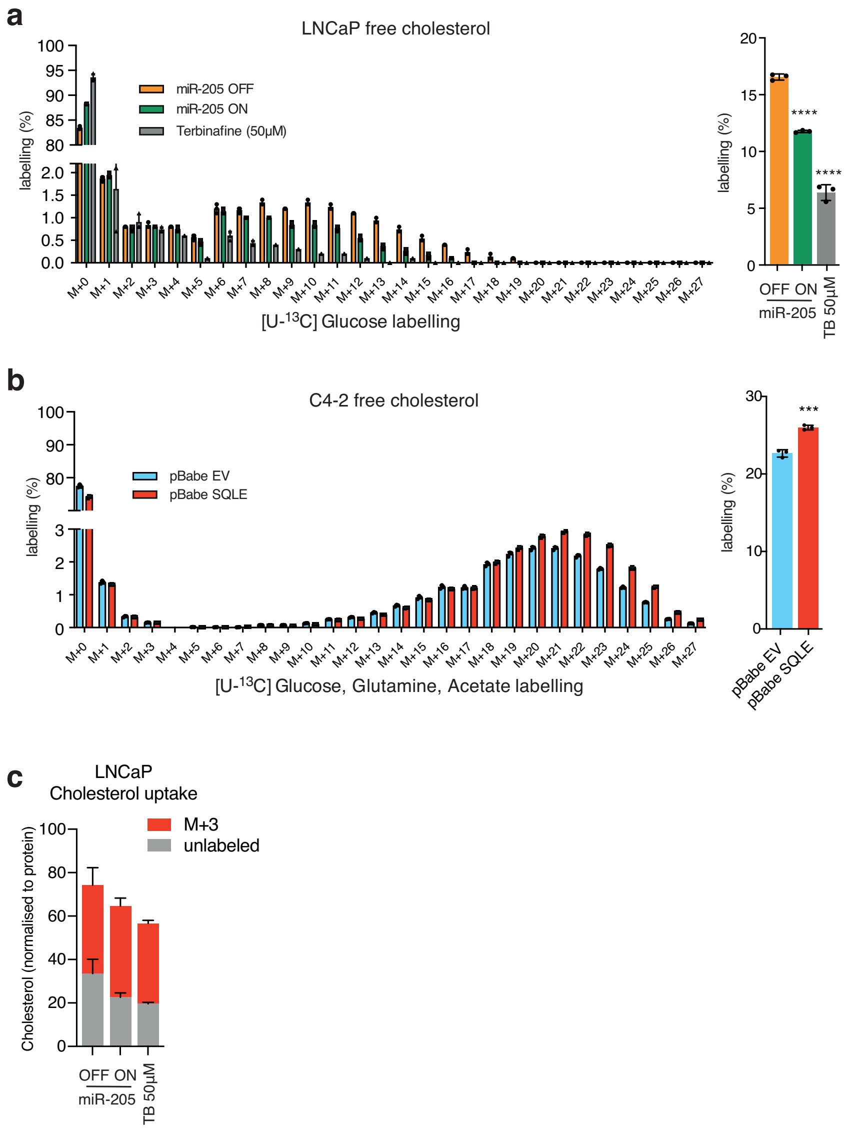

Extended Data Figure 3

Supplementary Figure 3:

a) LNCaP cells expressing doxycycline-inducible miR-205 were treated with 1 μg/mL of doxycycline for 72 hours (miR-205 ON) or solvent (MIR-205 OFF), or with 50 μM of terbinafine (TB) for 48 hours, and labelled with [U^{13}C]-glucose. Cells were extracted and analysed by LC-MS. Graphs show relative peak intensities of individual isotopologues (left graph) and overall labelled fractions (right graph) for free cholesterol. Data are presented as mean +/- SD of three independent biological replicates. (****p<0.0001, unpaired two-tailed Student's t-test).

b) C4-2 cells stably expressing SQLE (pBabe SQLE) and empty vector controls (pBabe EV) were labelled with [U^{13}C]-glutamine, [U^{13}C]-glucose and [U^{13}C]-acetate for 48 hours. Cells were extracted and analysed by LC-MS. Graphs show relative peak intensities of individual isotopologues (left graph) and overall labelled fractions (right graph) for free cholesterol. Data are presented as mean +/- SD of three independent biological replicates. (***p<0.001, unpaired two-tailed Student's t-test).

c) LNCaP cells were treated as in (a) and labelled with cholesterol-2,3,4-$^{13}C_3$ for 48 hours. Cells were extracted and analysed by LC-MS. Graphs show relative peak area normalised to protein for free cholesterol. The M+3 fraction represents cholesterol-2,3,4-$^{13}C_3$ taken up from the medium. Data are presented as mean +/- SD of three independent biological replicates.

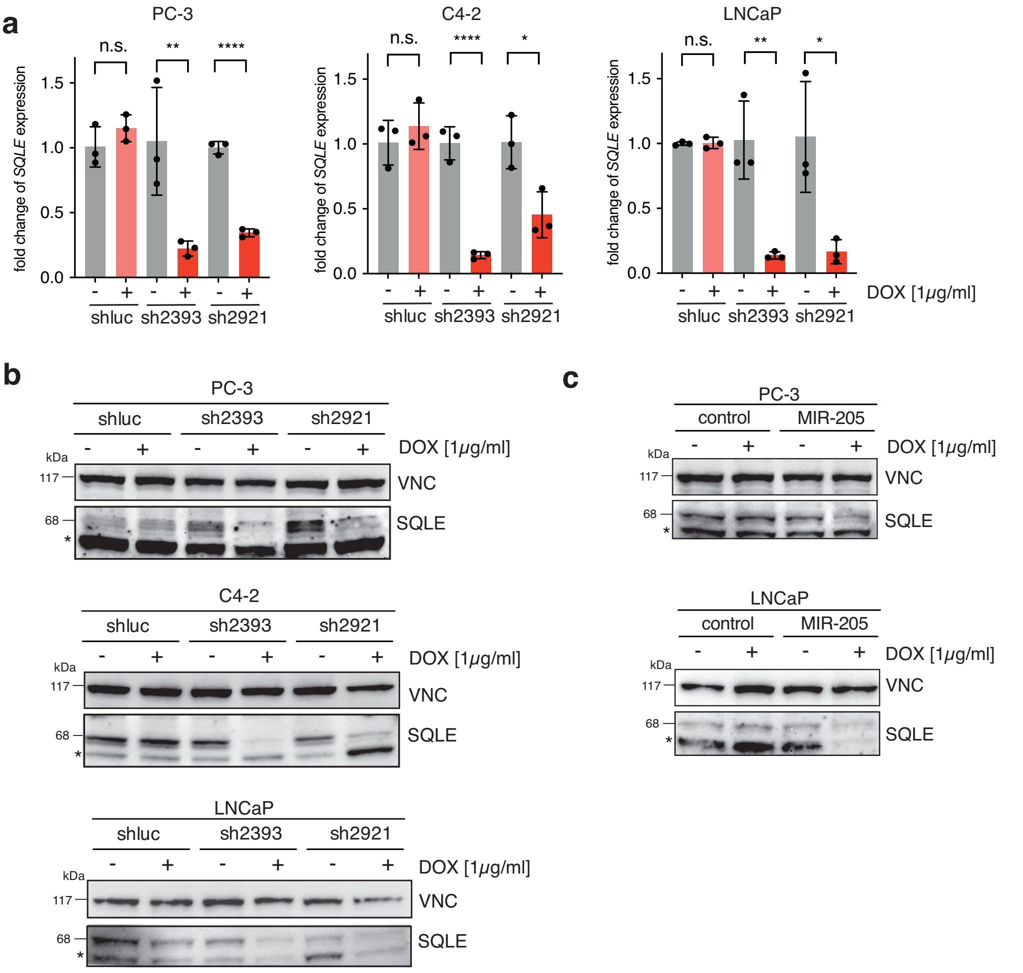

Extended Data Figure 4

Supplementary Figure 4:

a) qRT-PCR analysis of *SQLE* expression after silencing in PC-3, C4-2 and LNCaP cells expressing two independent shRNAs targeting *SQLE* (sh2393 or sh2921) or non-targeting shRNA (shluc) and treated with 1 µg/mL doxycycline (DOX) or solvent (EtOH) for 48 hours. Data presented as mean +/- SD of three independent samples (**p<0.01, ***p<0.001, ****p<0.0001, unpaired two-tailed Student's t-test).

b) WB showing expression of SQLE in PC-3 (top panel), C4-2 (middle panel) and LNCaP (bottom panel) cells treated as in (a). Vinculin (VNC) is shown as loading control (*unspecific band). A representative image of three independent experiments is shown.

c) WB showing expression of SQLE after doxycycline-induced miR-205 expression (DOX) in PC-3 and LNCaP cells compared to controls. Vinculin is shown as loading control (*unspecific band). A representative image of three independent experiments is shown.

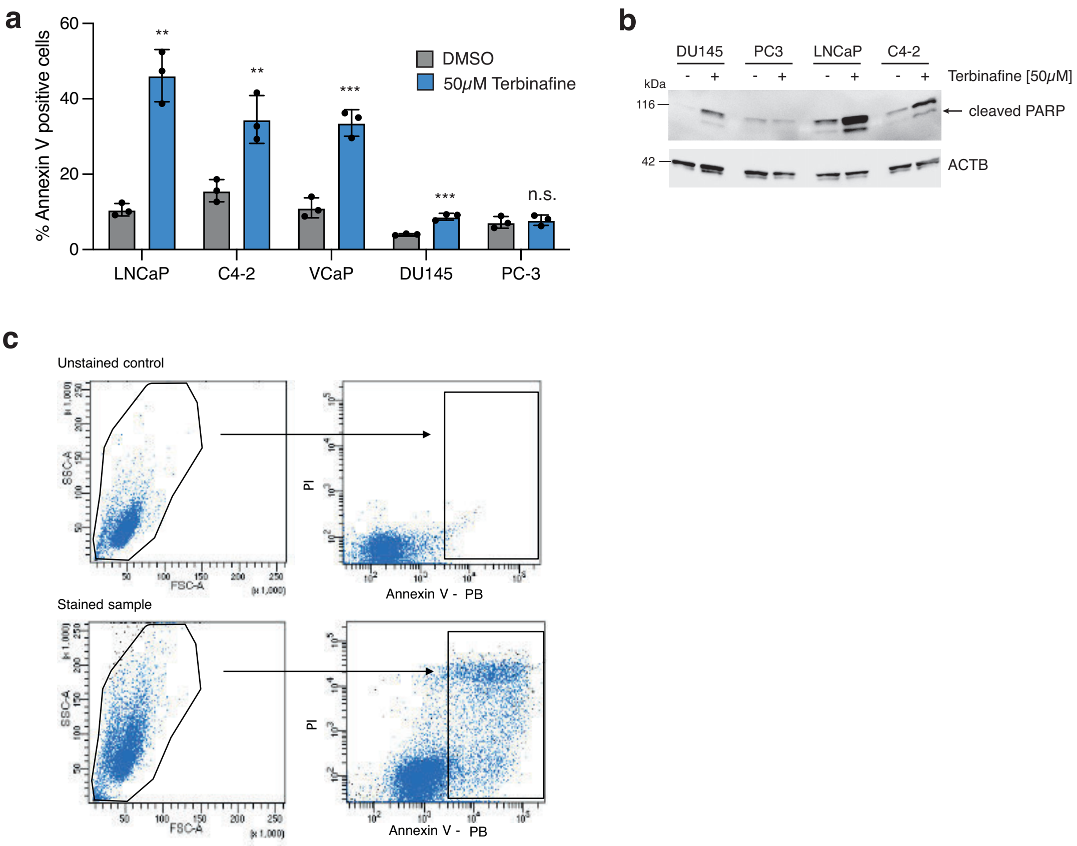

Extended Data Figure 5

Supplementary Figure 5:

a) LNCaP, C4-2, VCaP, DU-145 and PC-3 cells were treated with 50 μM of terbinafine for 96 hours. Apoptosis was detected using Annexin V staining and FACS analysis. Graph displays fraction of Annexin V positive cells. Data are presented as mean +/- SD of three independent samples (**p<0.01, ***p<0.001, n.s.= not significant, unpaired two-tailed Student's t-test).

b) Cells treated as in (a) were analysed for appearance of cleaved poly(ADP-ribose) polymerase 1 (PARP). Actin B is shown as loading control. A representative image of two independent experiments is shown.

c) Gating strategy used to identify Annexin V positive populations.

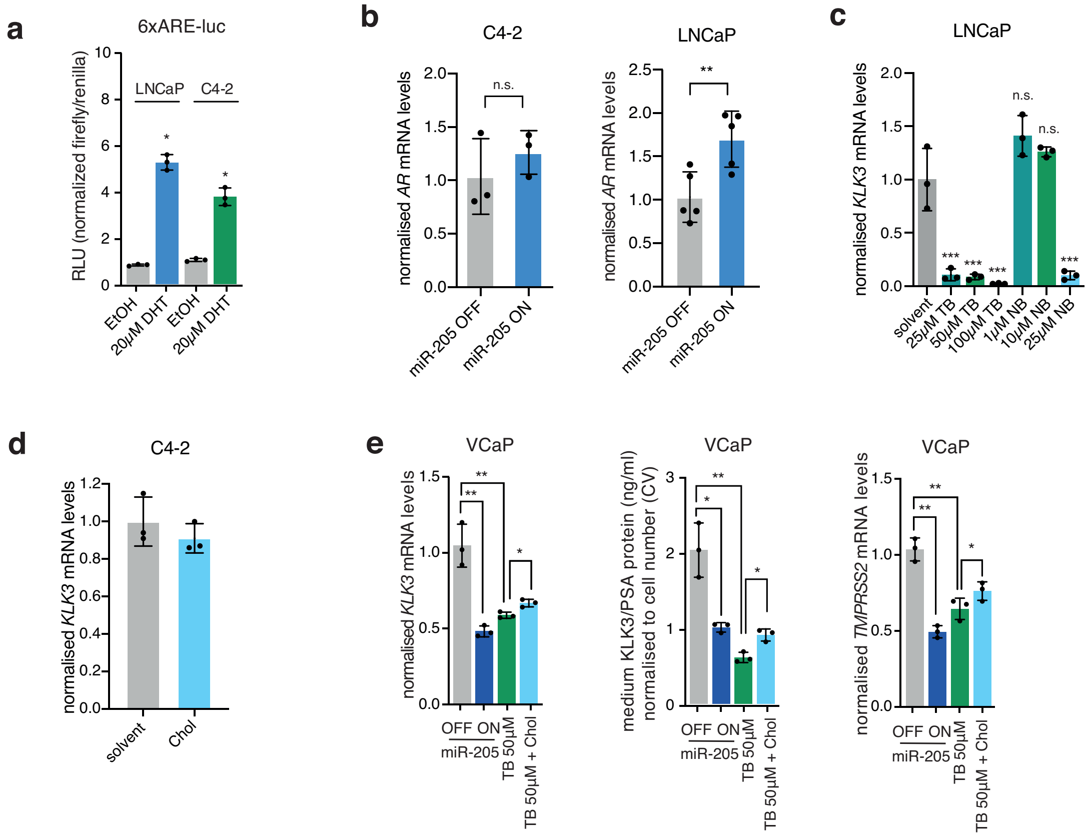

Extended Data Figure 6

Supplementary Figure 6:

a) Induction of the 6xARE-luc reporter in LNCaP or C4-2 cells after 12 hrs of treatment with dihydrotestosterone (DHT) as compared to vehicle controls. Graph shows relative light units (RLU) of firefly luciferase normalised to renilla luciferase (pRL-TK). Data are presented as mean +/- SD of three independent biological replicates (*p<0.05, unpaired two-tailed Student's t-test).

b) C4-2 and LNCaP cells expressing doxycycline-inducible miR-205 were treated with 1 µg/mL doxycycline (miR-205 ON) or solvent (miR-205 OFF) for 48 or 96 hours, respectively. Expression of *AR* mRNA was detected by qRT-PCR. Data are presented as mean +/- SD of three independent biological replicates (**p<0.01, n.s.= not significant, unpaired two-tailed Student's t-test).

c) LNCaP cells were treated with the indicated concentrations of terbinafine (TB) or NB-598 (NB) for 48 hours and analysed for expression of *KLK3* mRNA. Data show mean +/- SD of three independent biological replicates (**p<0.01, unpaired two-tailed Student's t-test).

d) C4-2 cell were treated with 0.125 µg/mL cell-permeable cholesterol for 48 hours. Expression of *KLK3* mRNA was detected by qRT-PCR. Data are presented as mean +/- SD of three independent biological replicates.

e) VCaP cells expressing doxycycline-inducible miR-205 were treated with 1 µg/mL doxycycline (miR-205 ON) or solvent (miR-205 OFF), or with 50 µM terbinafine (TB 50 µM) for 48 hours. Rescue experiments were carried out using 0.125 µg/mL of cell-permeable cholesterol (TB 50 µM + Chol). Expression of *KLK3* mRNA (left), levels of PSA in medium supernatants (middle) and expression of *TMPRSS2* mRNA were determined. Data show mean +/- SD of three independent biological replicates. (*p<0.05, **p<0.01, unpaired two-tailed Student's t-test).

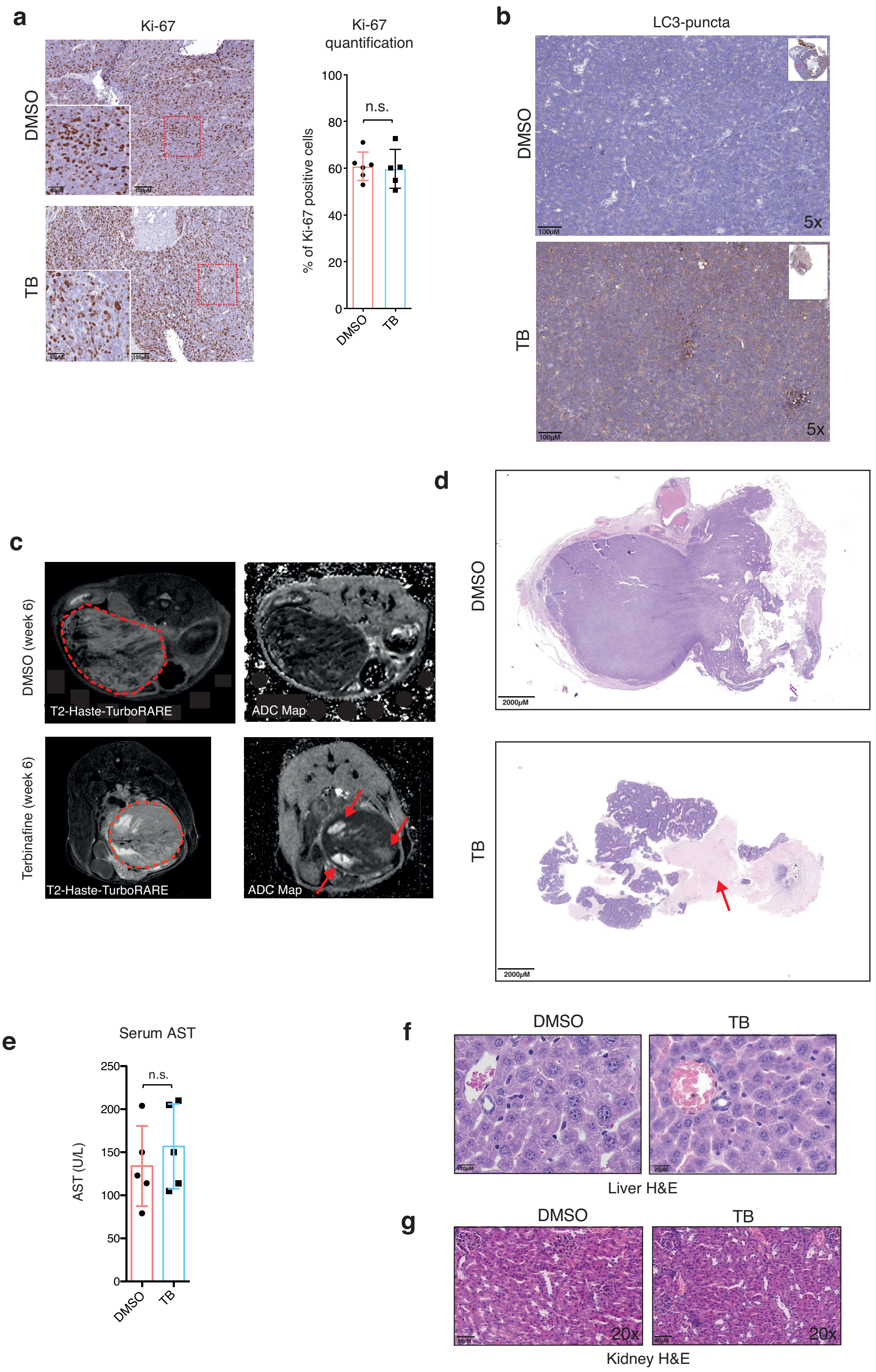

Extended Data Figure 7

Supplementary Figure 7:

a) Left: representative IHC sections stained for the proliferation marker Ki-67 in tumour bearing mice treated with vehicle (DMSO) or with 50 mg/kg terbinafine (TB) every 48 hours. Right: quantitative analysis of the percentage of Ki-67 positive cells per sample in tumours from vehicle and terbinafine treated mice using automated image analysis. Data are presented as mean +/- SD of DMSO (*N* = 6) and TB (*N* = 5) cohorts. (n.s.= not significant, unpaired two-tailed Student's t-test)

b) Overview images for LC3 puncta staining of tumour tissue from vehicle (DMSO) or terbinafine (TB) treated mice. Images are representative of DMSO (*N* = 6) and TB (*N* = 5) cohorts.

c) Murine MRI imaging. Shown are representative T2-weighted-(RARE) images and apparent diffusion coefficient (ADC) maps of vehicle (DMSO) or terbinafine (TB) treated mice at the indicated time point after orthotopic xenografting. Red arrows in terbinafine treated mice point to lacunae and diffusion disturbed areas in the tumour.

d) Representative overview hematoxylin and eosin (H&E) tumour sections of vehicle (DMSO) or terbinafine (TB) treated mice. Red arrow points to necrotic area in the terbinafine treated tumour. Images are representative of DMSO (*N* = 6) and TB (*N* = 5) cohorts.

e) Serum aspartate aminotransferase (AST) activity (U/L = units per litre) in vehicle (DMSO) or terbinafine (TB) treated mice. Data are presented as mean +/- SD of DMSO (*N* = 6) and TB (*N* = 5) cohorts. (n.s.= not significant, unpaired two-tailed Student's t-test).

f) H&E stained liver sections comprising portal fields of vehicle (DMSO) or terbinafine (TB) treated mice. Images are representative of DMSO (*N* = 8) and TB (*N* = 7) cohorts

g) H&E stained kidney sections of vehicle (DMSO) or terbinafine (TB) treated mice. Images are representative of DMSO (*N* = 8) and TB (*N* = 7) cohorts.

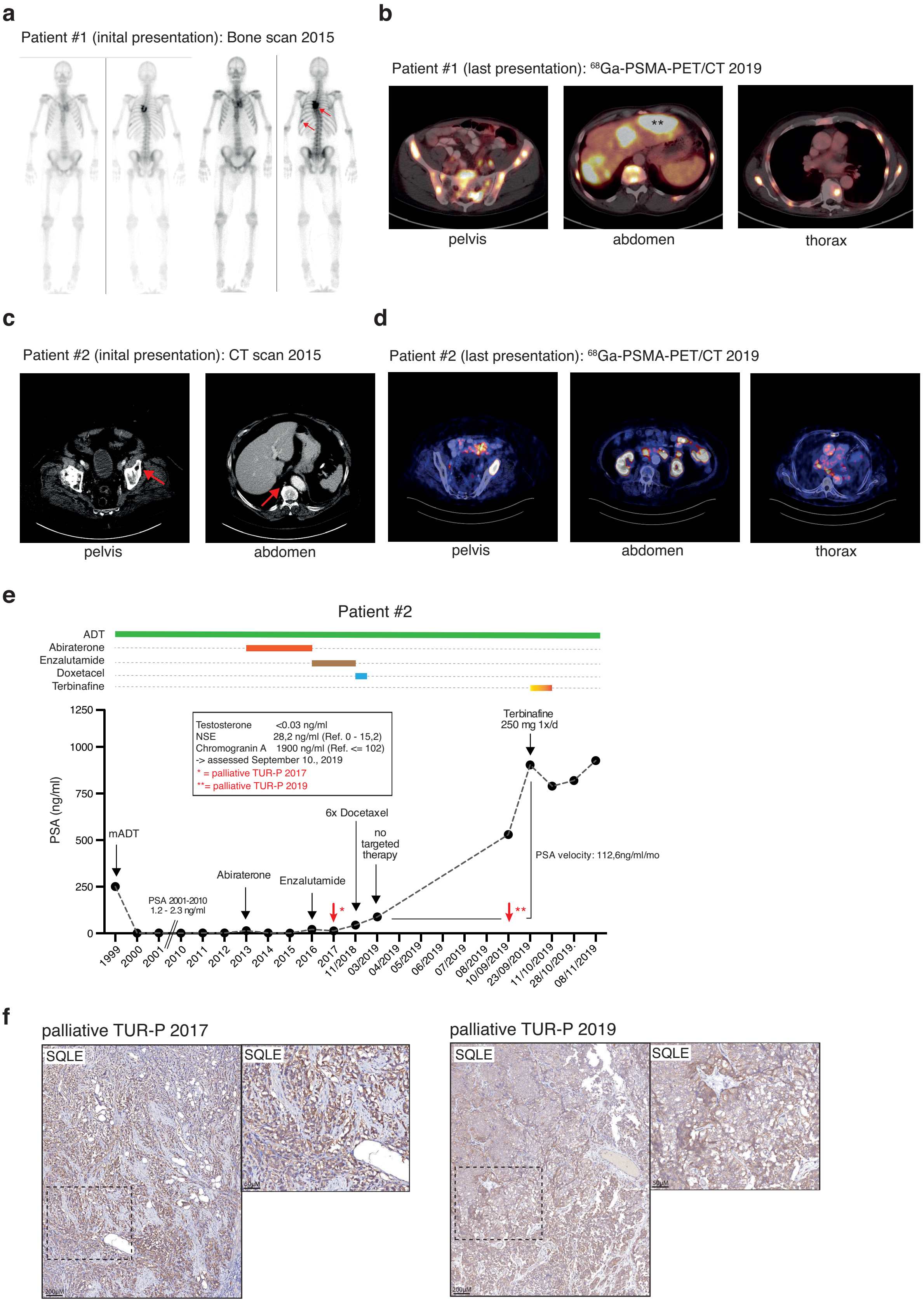

Extended Data Figure 8

Supplementary Figure 8:

a) Case description and initial presentation of patient #1 in 2011 (bone scan, red arrows point at osseous metastasis).

b) Last presentation of patient #1 in March 2019 (Ga^{68}PSMA-PET/CT with multiple osseous, lymph nodal and visceral metastases).

c) Case description and initial presentation of patient #2 in 2015 (CT scan, red arrows point at osseous and lymph nodal metastasis).

d) Last presentation of patient #2 in March 2019 (Ga^{68}PSMA-PET/CT with multiple osseous and lymph nodal metastases).

e) Serum PSA levels of patient #2 over the course of the disease. Respective treatment regimens are indicated above the graph. Inset: additional patient information. Red arrows indicate timepoints of tissue resection.

f) Representative IHC sections of tumour material from patient #2 stained for SQLE. Left: tumour tissue resected by desobstructive TUR-P in 2017. Right: tumour tissue by resected desobstructive TUR-P in 2019.
