# Supplementary material for: MiR-205-driven downregulation of cholesterol biosynthesis through SQLE-inhibition identifies therapeutic vulnerability in aggressive prostate cancer
Source: Nat Commun. 2021 Aug 20;12:5066. doi: 10.1038/s41467-021-25325-9 (PMC8379214; doi:10.1038/s41467-021-25325-9)
Supplement: Supplementary file 3 — Description of Additional Supplementary Files [file 41467_2021_25325_MOESM3_ESM.pdf]

Description of Additional Supplementary Files

File Name: Supplementary Software 1
Description: Commented custom R script for TCGA prostate cancer data extraction and processing with data analysis example
