# Supplementary material for: MiR-205-driven downregulation of cholesterol biosynthesis through SQLE-inhibition identifies therapeutic vulnerability in aggressive prostate cancer
Source: Nat Commun. 2021 Aug 20;12:5066. doi: 10.1038/s41467-021-25325-9 (PMC8379214; doi:10.1038/s41467-021-25325-9)
Supplement: Supplementary file 4 — Supplementary Software 1 [file 41467_2021_25325_MOESM4_ESM.pdf]

Supplementary Software 1 - Kalogirou et al.

MiR-205-driven downregulation of cholesterol biosynthesis through SQLE-inhibition identifies therapeutic vulnerability in aggressive prostate cancer

R-Script for TCGA data analysis

```
##load packages - TCGA prostate RNASeqV2 and plyr
library(TCGA2STAT)
library(plyr)

##import TCGA PRAD data along with clinical parameters of interest
rna.pca <- getTCGA(disease = "PRAD", data.type = "RNASeq2",
type="RPKM",clinical=TRUE,cvars=c("gleasonscore","psavalue","daystopsa","pathology
Tstage","pathologyNstage"))

## exclude data with missing values, regroup, rename, assign numeric
newdata<-rna.pca$merged.dat
head(newdata[,1:5])
newdata<-newdata[-c(6,145,203,370,382,391),]
rnadata<-newdata[1:6]
rnadata$GLEASONSCORE<-as.numeric(rnadata$GLEASONSCORE)
rnadata$PSAVALUE<-as.numeric(rnadata$PSAVALUE)
rnadata$DAYSTOPSA<-as.numeric(rnadata$DAYSTOPSA)
rnadata$fu_months<-rnadata$DAYSTOPSA/30

#convert t-stages into groups
rnadata$PATHOLOGYTSTAGENUMERIC<-revalue(rnadata$PATHOLOGYTSTAGE, c("t2a"="2",
"t2b"="2", "t2c"="2","t3a"="3","t3b"="3","t4"="4"))

##divide into organ-confined PCa
rnadata$stagegroup<-ifelse(rnadata$PATHOLOGYTSTAGENUMERIC<=2,"0", "1")

## convert n-stages into nodal-positive/negative
rnadata$nodegroup<-revalue(rnadata$PATHOLOGYNSTAGE, c("n0"="0", "n1"= "1"))

##divide into low and high-risk PCa according to gleason score
rnadata$gleasongroup<-ifelse(rnadata$GLEASONSCORE<=8,"0", "1")

#integrate gene of interest - SQLE
x<-newdata["SQLE"]
newx<-unlist(x,use.names=FALSE)
numericx<-as.numeric(newx)
rnadata$expression<-numericx

## convert values of interest into numeric
rnadata$relaps<-as.numeric(rnadata$relaps)
rnadata$group<-as.numeric(rnadata$group)

## clinical parameter analysis example - gleason score
boxplot(expression~GLEASONSCORE, ylim=c(0,5000),data=rnadata)
##check on expression
boxplot(expression~gleasongroup, ylim=c(0,5000), data=rnadata)
##ttest
t.test(expression ~ gleasongroup, data = rnadata)
```

Legend: Commented custom R script for TCGA prostate cancer data extraction and processing with data analysis example.
